# Efficacy and Safety of Sodium Tanshinone IIA Sulfonate Injection on Hypertensive Nephropathy: A Systematic Review and Meta-Analysis

**DOI:** 10.3389/fphar.2019.01542

**Published:** 2019-12-24

**Authors:** Junyao Xu, Chenghua Zhang, Xiaoqing Shi, Jie Li, Ming Liu, Weimin Jiang, Zhuyuan Fang

**Affiliations:** Institute of Hypertension, Affiliated Hospital of Nanjing University of Chinese Medicine, Nanjing, China

**Keywords:** sodium tanshinone IIA sulfonate injection, hypertensive nephropathy, systematic review, meta-analysis, efficacy, safety

## Abstract

**Background:** Sodium tanshinone IIA sulfonate (STS) injection, the extractive of traditional Chinese medicine Danshen, is supposed to be a supplementary treatment in hypertensive nephropathy.

**Objectives:** To evaluate the efficacy and safety of STS in treatment of hypertensive nephropathy.

**Methods:** We systematically searched China National Knowledge Infrastructure (CNKI), Chinese Scientific Journals Database (VIP), Wan-fang database, Chinese Biomedicine Database (CBM), PubMed, Embase, Web of Science, and Cochrane Library from their inception to December 2018. All studies were screened by two reviewers according to the inclusion and exclusion criteria independently. The Cochrane Collaboration's risk tool was used to assess the methodological quality of the included studies. Reviewer Manager 5.3 was employed for statistical analysis.

**Results:** Sixteen trials involving 1,696 patients were included. The meta-analysis results indicated a combination of STS and angiotensin receptor blockers (ARBs) was more effective than ARB monotherapy in modulating hypertensive nephropathy, as represented by improved estimated glomerular filtration rate (eGFR) [mean difference (MD) = 6.87, 95% CI (4.47, 9.28), *P* < 0.00001] and reduced 24 h urinary protein [MD = −0.23, 95% CI (−0.27, −0.19), *P* < 0.00001], serum creatinine (SCr) [MD = −21.74, 95% CI (−24.11, −19.38), *P* < 0.00001], cystatin-C [MD = −0.16, 95% CI (−0.24, −0.07), *P* = 0.0003], urinary immunoglobulin G (IgG) [MD = −0.85, 95% CI (−1.11, −0.59), *P* < 0.00001], and urinary transferrin [MD = −0.61, 95% CI (−1.04, −0.17), *P* = 0.007]. In addition, the combination therapy had better control in systolic blood pressure (SBP) [MD = −6.53, 95% CI (−8.19, −4.87), *P* < 0.00001] and diastolic blood pressure (DBP) [MD = −4.14, 95% CI (−5.69, −2.59), *P* < 0.00001]. Only three trials reported adverse events, and no adverse drug reactions were observed.

**Conclusions:** STS combined with ARBs had a stronger effect on improving renal function in patients with primary hypertensive nephropathy than ARB monotherapy. The combination therapy also provided auxiliary hypotensive effects. Further large-scale, multicenter, and rigorously designed randomized controlled trials (RCTs) should be conducted to confirm our findings.

## Introduction

The prevalence of primary hypertension (PH), a chronic disease, is increasing worldwide. It is estimated that approximately one of three people suffer from high blood pressure (BP) all over the world (Lim et al., 2012). PH has been one of the leading risk factors for global disease burden (Whelton et al., 2018). Although a system therapeutic regimen has been established, hypertension still remains undertreated and uncontrolled especially in developing countries like China (Lu et al., 2017). Patients with hypertension usually have a series of secondary lesions. The kidney, as a vital organ in balancing the volume load and the toxin level, is extremely vulnerable to high BP. It is known that structural damage to the renal unit, such as the barotrauma damage to the glomerular filtration barrier, can lead to compensatory but maladaptive increases in the reuptake of water and sodium by the renal tubule, further exacerbating BP abnormalities (Bidani et al., 2013; Hall et al., 2014). Nowadays, hypertensive kidney disease has been the second leading cause of end-stage renal disease (ESRD) after diabetes mellitus (Hart and Bakris, 2010). Approximately 30,000 individuals in the United States are diagnosed with hypertension-associated ESRD yearly, which exerts substantial adverse influence on public health and health-care financing (Suneel et al., 2011).

BP control is regarded as a vital target in patients of hypertensive nephropathy. Agents that inhibit the renin–angiotensin–aldosterone system (RAAS) are the first-line drugs of choice for those with hypertensive nephropathy since they can effectively reduce BP, proteinuria, and chronic kidney disease (CKD) progression as well as cardiovascular events associated with hypertension, diabetes, and vascular diseases (The GISEN Group, 1997; Lewis et al., 2001; Brenner et al., 2001). However, existing data have demonstrated that well controlled blood pressure (<130/80 mmHg) slows but does not stop progression of renal injury (Suneel et al., 2011). Furthermore, use of these drugs alone at the recommended dosages for BP control is usually unable to achieve enough renoprotective effect. Thus, in addition to BP control, nephropathy protection is necessary.

Traditional Chinese medicine is the most common form of both alternative and complementary medicine in Asia which has been used extensively for the treatment of CKD (Huang et al., 2018). Danshen, the dried root and rhizome of *Salvia miltiorrhiza Bge* (Labiatae), is a popular medicinal herb that has long been used for the treatment of various diseases. Studies have shown that Danshen is the top single herb prescribed for the treatment of CKD in outpatients in China (Huang et al., 2018). Sodium tanshinone IIA sulfonate (STS) injection, the extract of Danshen, has been widely used in current clinical practice for its activities in anti–free radical induced tissue damage, arteriolar vasodilation, lowering blood viscosity, and so on (Bai, 2012). It mainly contains STS (sodium 1,6,6-trimethyl-10, 11-dioxo-6,7,8,9,10,11- hexahydrophenanthro[1,2-b]furan-2-sulfonate) (Hao et al., 2007; Chen et al., 2013). The concentration of the commercial injection is 5 mg/ml, and the purity is more than 90% (STS: 90%–98%) (Wang et al., 2006; Liu, 2010; Chen et al., 2013; Dong et al., 2019). Quality control and chemical analyses of the material were also reported. (Wang et al., 2006; Hao et al., 2007; Liu, 2010; Chen et al., 2013; Dong et al., 2019). Procedures are fully reproducible.

In recent years, a large number of clinical trials suggested that STS and angiotensin receptor blocker (ARB) combination benefited patients with hypertensive nephropathy. However, no definite conclusion was drawn on this. Therefore, we conducted this systematic review and meta-analysis to investigate the efficacy of STS as an adjuvant agent in the management of hypertensive nephropathy.

## Materials and Methods

Readers can access the protocol of this systematic review in International Prospective Register of Systematic Reviews (PROSPERO) (CRD42018114511).

### Database and Search Strategies

A literature search was carried out in the following eight databases from their inception to December 2018: China National Knowledge Infrastructure (CNKI), Chinese Scientific Journals Database (VIP), Wan-fang database, Chinese Biomedicine Database (CBM), PubMed, Embase, Web of Science, and Cochrane Library. The publishing language was restricted to Chinese and English. Search terms including “hypertension,” “sodium tanshinone IIA sulfonate injection,” “tanshinone IIA,” “kidney diseases,” “renal insufficiency,” “kidney failure, chronic,” “hypertensive nephropathy,” “kidney injury,” “kidney damage,” “renal damage,” “renal injury,” “nephrosclerosis,” “renal impairment,” and “kidney impairment” were used individually or in combination. In addition, a filter for clinical trials was also employed.

### Inclusion Criteria

Studies meeting the following criteria were included: (1) randomized controlled trials (RCTs), regardless of blinding or publication types; (2) patients with hypertensive nephropathy, (i) meeting the diagnostic criteria of hypertension, (ii) presenting with clinical features of renal injury like persistent proteinuria and increasing levels of serum creatinine (SCr), and (iii) without primary renal disease, secondary hypertension, or some other conditions such as diabetes mellitus causing renal injury; (3) intervention using STS combined with ARBs compared to ARB monotherapy; and (4) assessment of 24 h urinary protein as the primary outcome plus at least one additional outcome measure, which could be SCr, estimated glomerular filtration rate (eGFR), urinary immunoglobulin G (IgG), cystatin-C (Cys-C), urinary transferrin, systolic blood pressure (SBP) reduction, or diastolic blood pressure (DBP) reduction.

### Exclusion Criteria

Studies were excluded if they met the following criteria: (1) duplicated publications; (2) non-clinical research, basic research, and review articles as well as case reports and theoretical discussions; (3) use of any other western medicines and/or herbal medicines during the research; or (4) outcome data of interest were unavailable for meta-analysis.

### Data Extraction

Two investigators (JX, CZ) independently screened and extracted the data according to the inclusion and exclusion criteria. After the general details, patients' characteristics, interventions, and outcomes were extracted, a cross-check was then done. Any disagreements were resolved through discussion or the verification of a third investigator (XS).

### Quality Assessment

Evaluation of methodological quality of the included studies was conducted by the same two investigators (JX, CZ), who used the Cochrane Collaboration's risk tool. Random sequence generation (selection bias), allocation concealment (selection bias), blinding of participants and personnel (performance bias), blinding of outcome assessment (detection bias), incomplete outcome date (attrition bias), selective reporting (reporting bias), and other bias were assessed. The grade of bias risk was classified as “low,” “high,” and “unclear.” We also performed a funnel plot to evaluate publication bias.

### Data Synthesis and Analysis

Revman 5.3 software was employed to pool the effect size. Mean difference (MD) or standardized mean difference (SMD) and 95% confidence intervals (CI) were used for continuous variables. Heterogeneity was evaluated statistically using the χ^2^ test and inconsistency index statistic (*I*^2^) (Higgins et al., 2003). If substantial heterogeneity existed (*I^2^* > 50% or *P* < 0.05), a random effect model was applied; otherwise, we adopted a fixed effect model (DerSimonian and Laird, 1986). We also performed subgroup analysis and sensitivity analysis to explore the potential sources of heterogeneity and inspect the stability of the result.

## Results

### Search Results

**Figure 1:**
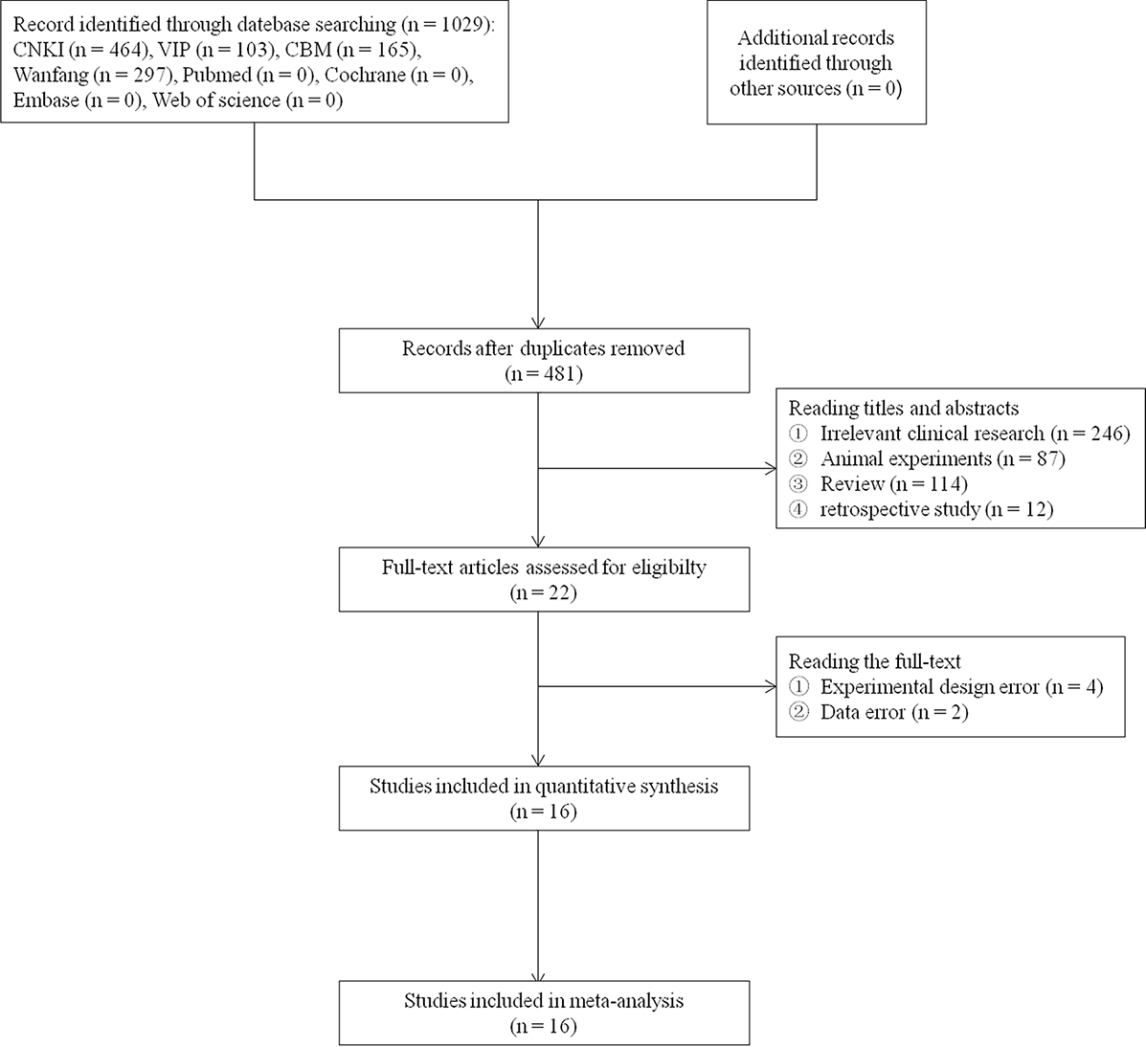
Flow diagram of the literature selection.

A total of 1,029 articles were initially obtained through the search strategy. After excluding 548 duplications, the remaining articles were screened based on their titles and abstracts, and 459 records were removed. Out of the remaining 22 articles assessed for eligibility, 16 articles met the eligibility criteria of this systematic review and meta-analysis (Zhu et al., 2011; Yang et al., 2012; Wu, 2013; Zou et al., 2013; Cao et al., 2014; Li, 2014; Yu, 2014; Liu, 2015; Luo, 2015; Qiu, 2015; Zhu, 2015; Bi et al., 2016; Li, 2016; Wang et al., 2016; Li and Guo, 2017; Tusonguri, 2017). The flowchart for literature retrieval is shown in **Figure 1**.

### Study Characteristics

All 16 included trials were conducted in China from 2011 to 2017 and involved a total of 1,696 participants. Sample size ranged from 50 to 230, and only seven trials included more than 100 participants (Li, 2014; Luo, 2015; Qiu, 2015; Bi et al., 2016; Li, 2016; Wang et al., 2016; Tusonguri, 2017). The average age of the enrolled participants was 63, with 55.5% male patients. The intervention used in all experimental groups was STS combined with ARBs (valsartan, V; irbesartan, Ir; losartan, L), and the control treatment was ARBs. The dose range of STS was from 40 to 60 mg/d. The duration of treatment was 2 or 4 weeks. More details of the included studies are presented in **Table 1**.

**Table 1:** Characteristics of included studies.

Study	Sex (M/F)	Case (E/C)	Age (E/C) (years)	Course of disease (E/C) (years)	Therapy of experiment group	Therapy of control group	Durance (weeks)	Outcomes
Tusonguri, 2017	68/60	64/64	39–81	3–22	TI 60 mg q.d.+V 80 mg q.d.	V 80 mg q.d.	2	(1) (2) (3) (4) (7)	
Li and Guo, 2017	104/96	100/100	63.5 ± 8/64.1 ± 8.1	5.1 ± 1.8/5.8 ± 1.9	TI 60 mg q.d.+V 200 mg q.d.	V 200 mg q.d.	2	(1) (2) (3) (4) (7)	
Wang et al., 2016	69/61	65/65	58.4 ± 2.9/57.4 ± 3.2	6.3 ± 1.4/6.4 ± 1.2	TI 60 mg q.d.+V 80 mg q.d.	V 80 mg q.d.	2	(1) (2) (3) (7)	
Li, 2016	35/25	30/30	23–70	Unclear	TI 60 mg q.d.+V 80 mg q.d.	V 80 mg q.d.	4	(1) (2) (3) (4) (5) (6) (8)	
Bi et al., 2016	Unclear	50/50	41–62	11.3 ± 5.1/12.6 ± 6.1	TI 50 mg q.d.+Ir 150 mg q.d.	Ir 150 mg q.d.	4	(1) (2) (3) (6) (9) (10)	
Zhu, 2015	55/43	49/49	40–75	1–8	TI 60 mg q.d.+V 80 mg q.d.	V 80 mg q.d.	2	(1) (2) (3)	
Qiu, 2015	62/46	54/54	43–71	5–20	TI 60 mg q.d.+V 80 mg q.d.	V 80 mg q.d.	2	(1) (2) (3) (4) (6) (7) (8)	
Luo, 2015	82/38	60/60	35–81	10 ± 8/10 ± 9	TI 50 mg q.d.+V 80 mg q.d.	V 80 mg q.d.	2	(1) (2) (3) (4) (7)	
Liu, 2015	53/37	45/45	27–65	1.5–12	TI 50 mg q.d.+L 50 mg q.d.	L 50 mg q.d.	2	(1) (2) (3) (4) (5)	
Yu, 2014	45/22	34/33	42–84	4–16	TI 60 mg q.d.+V 80 mg q.d.	V 80 mg q.d.	2	(1) (2) (3) (4) (7)	
Li, 2014	123/107	115/115	40–75	4–10	TI 60 mg q.d.+V 80 mg q.d.	V 80 mg q.d.	2	(1) (2) (3) (4) (7)	
Cao et al., 2014	44/45	45/44	63.1 ± 16.8/62.3 ± 17.6	12.4 ± 6.2/10.2 ± 8.0	TI 60 mg q.d.+Ir 150 mg q.d.	Ir 150 mg q.d.	4	(1) (2) (3) (6) (11) (12)	
Zou et al., 2013	45/35	40/40	62 ± 10/63 ± 8	10 ± 8/10 ± 6	TI 60 mg q.d.+V 80 mg q.d.	V 80 mg q.d.	2	(1) (2) (3) (4) (7)	
Wu, 2013	27/23	25/25	43.4 ± 7.3	91.3 ± 16.8 (months)	TI 40 mg q.d.+L 50 mg q.d.	L 50 mg q.d.	2	(3) (4) (5) (15)	
Yang et al., 2012	42/44	43/43	63.1 ± 16.8/62.3 ± 17.6	12.4 ± 6.2/10.2 ± 8.0	TI 60 mg q.d.+V 80 mg q.d.	V 80 mg q.d.	4	(1) (2) (3) (6) (8) (12) (13)	
Zhu et al., 2011	31/29	30/30	52.1 ± 8.6	89.1+17.3 (months)	TI 40 mg q.d.+L 50 mg q.d.	L 50 mg q.d.	2	(1) (2) (3) (4) (5) (14) (15)	

M, male; F, female; E, experimental group; C: control group; TI, tanshinone injection; V, valsartan; Ir, irbesartan; L, losartan; q.d., once daily; (1) systolic blood pressure; (2) diastolic blood pressure; (3) 24 h urinary protein; (4) serum creatinine; (5) estimated glomerular filtration rate; (6) urinary IgG; (7) cystatin-C; (8) urinary transferrin; (9) podocalyxin; (10) carboxyterminal propeptide of type I procollagen; (11) urinary β2-microglobulin; (12) microalbuminuria; (13) urinary alpha1-microglobulin; (14) mean arterial pressure; (15) N-acetyl-beta-D-glucosaminidase.

### Quality Assessment of Included Studies

**Figure 2:**
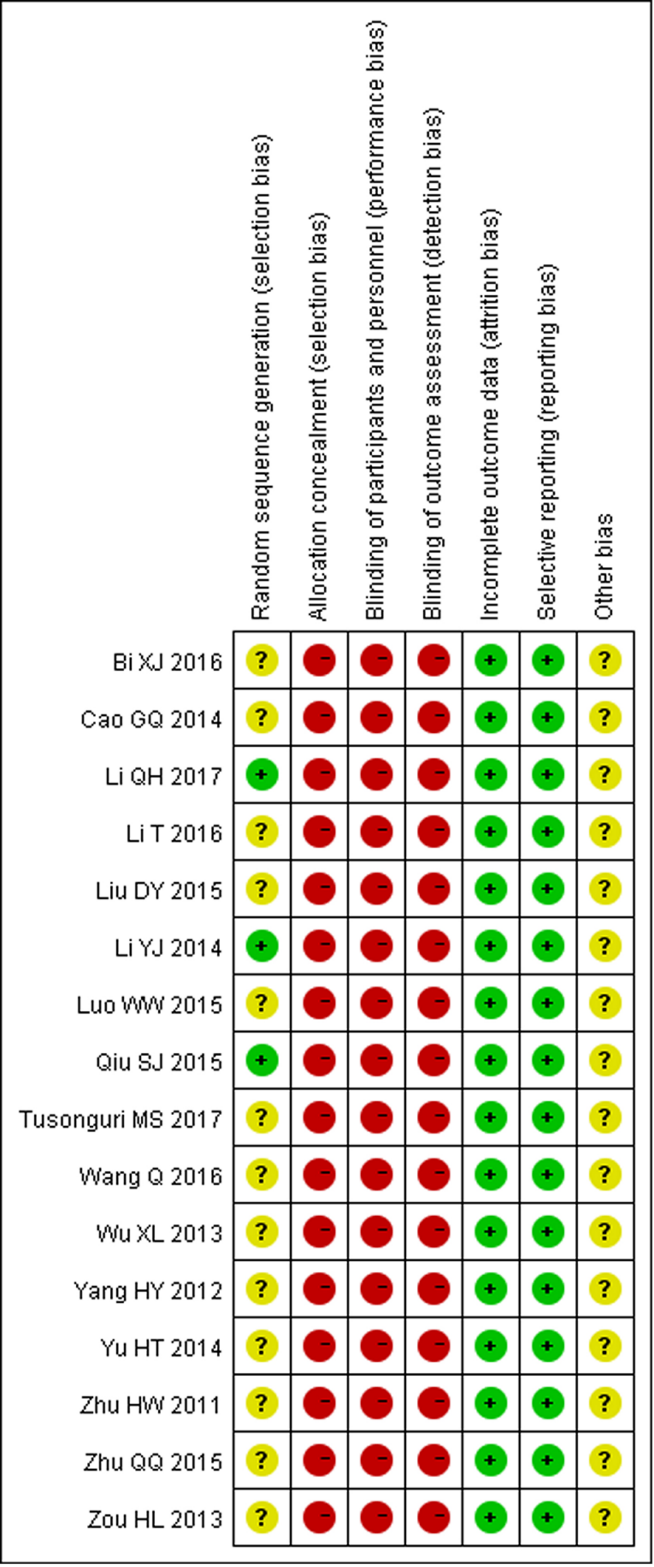
Risk of bias summary.

**Figure 3:**
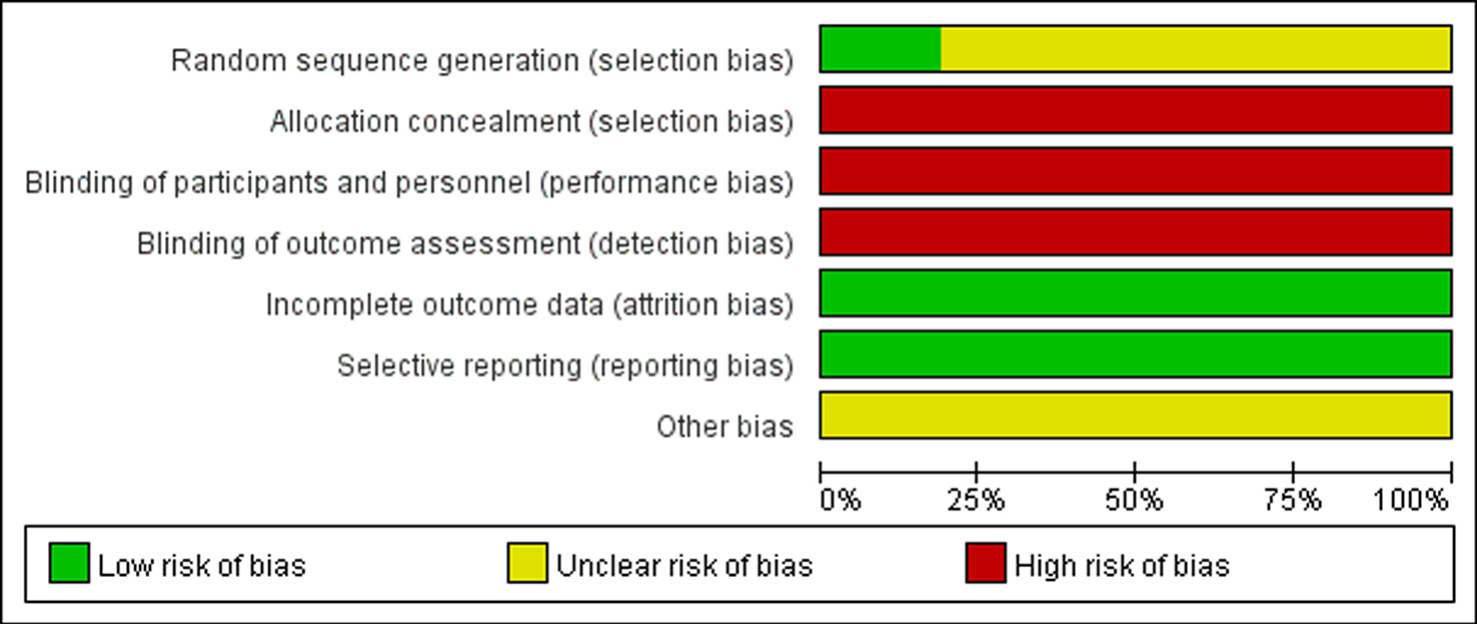
Risk of bias graph of included trials.

The methodological quality assessments of all included studies are shown in **Figures 2** and **3**. Only three trials described the randomization method used in their studies, while others did not report any specific randomization technique (Li, 2014; Qiu, 2015; Li and Guo, 2017). None of studies reported allocation concealment procedure and blinding. In general, most of the included studies had high risk of bias and low methodological quality.

## Meta-Analysis Results

### 24 h Urinary Protein (g/d)

**Figure 4:**
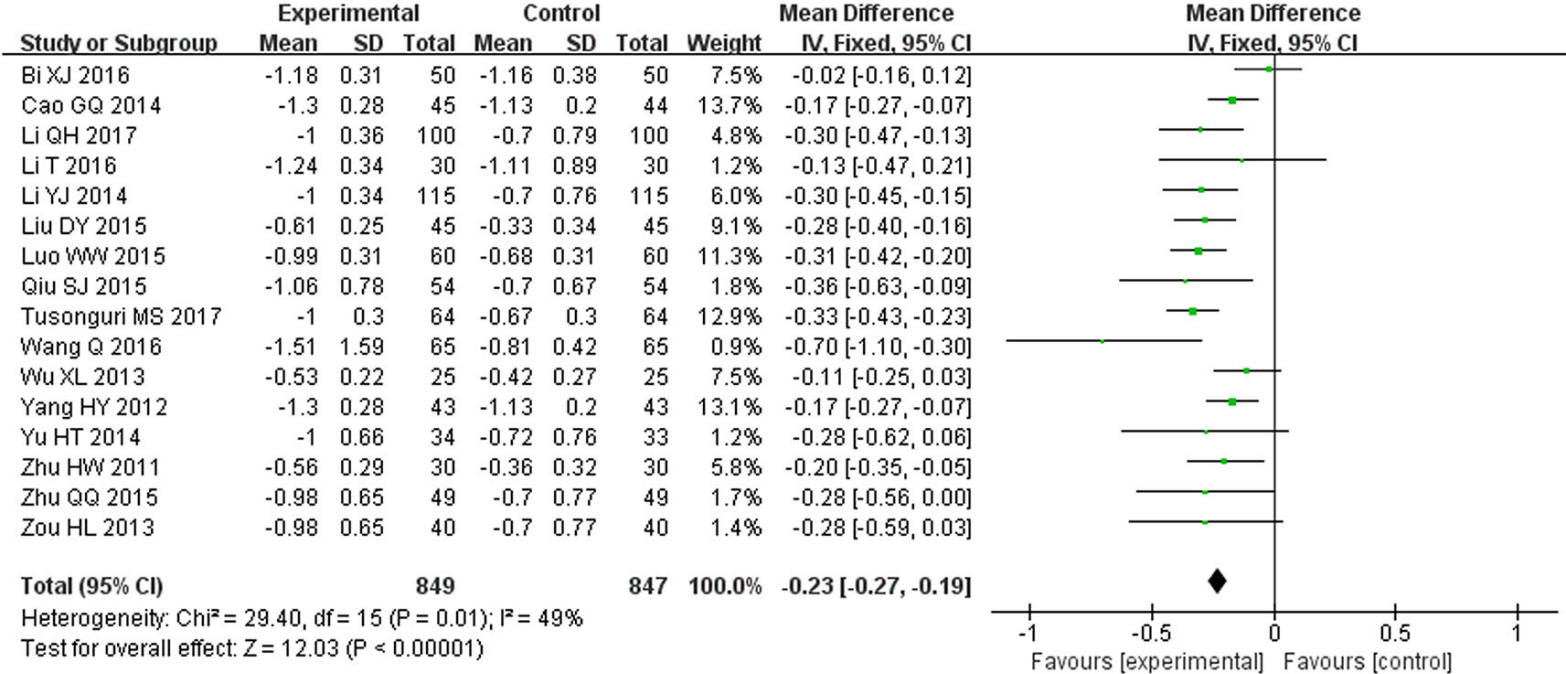
Meta-analysis for comparison of 24 h urinary protein levels between the experimental and control groups.

All 16 trials reported 24 h urinary protein measurement results. Following the test of heterogeneity (*P* = 0.01, *I^2^* = 49%), we used a fixed effect model. The pooled analysis indicated that the intervention of STS combined with ARBs had better effect in decreasing 24 h urinary protein. The difference between two groups was statistically significant (MD = −0.23, 95% CI: −0.27 to −0.19; *P* < 0.00001, **Figure 4**). This result suggests that STS as an adjunct therapy to ARBs may alleviate renal injury more effectively than ARBs alone.

### Serum Creatinine (μmol/L)

**Figure 5:**
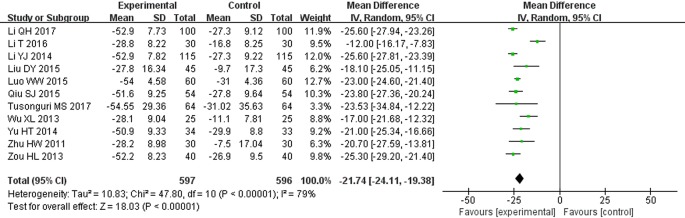
Meta-analysis for comparison of serum creatinine (SCr) levels between the experimental and control groups.

Eleven clinical trials reported SCr levels (Zhu et al., 2011; Wu, 2013; Zou et al., 2013; Li, 2014; Yu, 2014; Liu, 2015; Luo, 2015; Qiu, 2015; Li, 2016; Li and Guo, 2017; Tusonguri, 2017). Following the test of heterogeneity (*P* < 0.00001, *I^2^* = 79%), a random effect model was used to estimate pooled effect size. The results showed that patients receiving the therapy of STS combined with ARBs had significantly decreased SCr compared with control groups. The difference between two groups was statistically significant (MD = −21.74, 95% CI: −24.11 to −19.38; *P* < 0.00001, **Figure 5**).

### Estimated Glomerular Filtration Rate (ml/Min·1.73m^2^)

**Figure 6:**
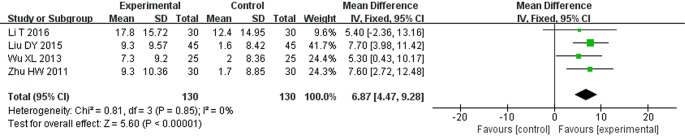
Meta-analysis for comparison of estimated glomerular filtration rate (eGFR) levels between the experimental and control groups.

There were four studies that reported eGFR results (Zhu et al., 2011; Wu, 2013; Liu, 2015; Li, 2016). A fixed effect model was adopted to pool the data because of no heterogeneity found in these studies (*P* = 0.85, *I^2^* = 0%). The results showed that the therapy of STS combined with ARBs was more effective in maintaining eGFR (MD = 6.87, 95% CI: 4.47, 9.28, *P* < 0.00001, **Figure 6**).

### Cystatin-C (mg/L)

**Figure 7:**
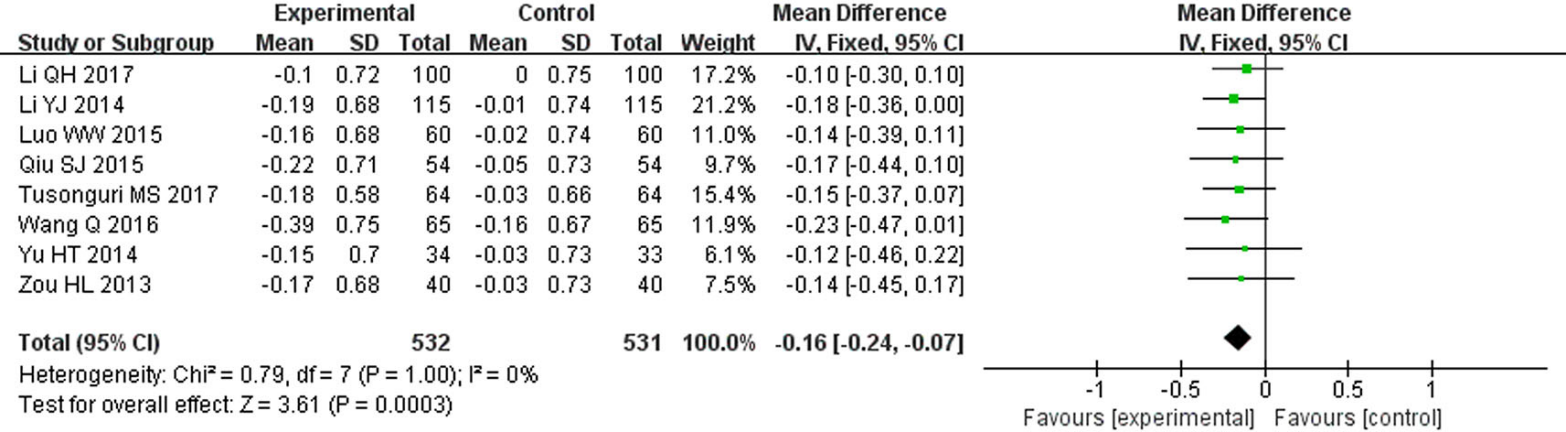
Meta-analysis for comparison of cystatin-C (Cys-C) levels between the experimental and control groups.

There were 8 studies that showed Cys-C results (Zou et al., 2013; Li, 2014; Yu, 2014; Luo, 2015; Qiu, 2015; Wang et al., 2016; Li and Guo, 2017; Tusonguri, 2017). A fixed effect model was adopted to pool the data because of no heterogeneity found in these studies (*P* = 1.0, *I^2^* = 0%). The results revealed that STS plus ARBs was superior to ARBs alone in reducing Cys-C (MD = −0.16, 95% CI: −0.24 to −0.07; *P* = 0.0003, **Figure 7**).

### Urinary Immunoglobulin G (mg/dl)

**Figure 8:**
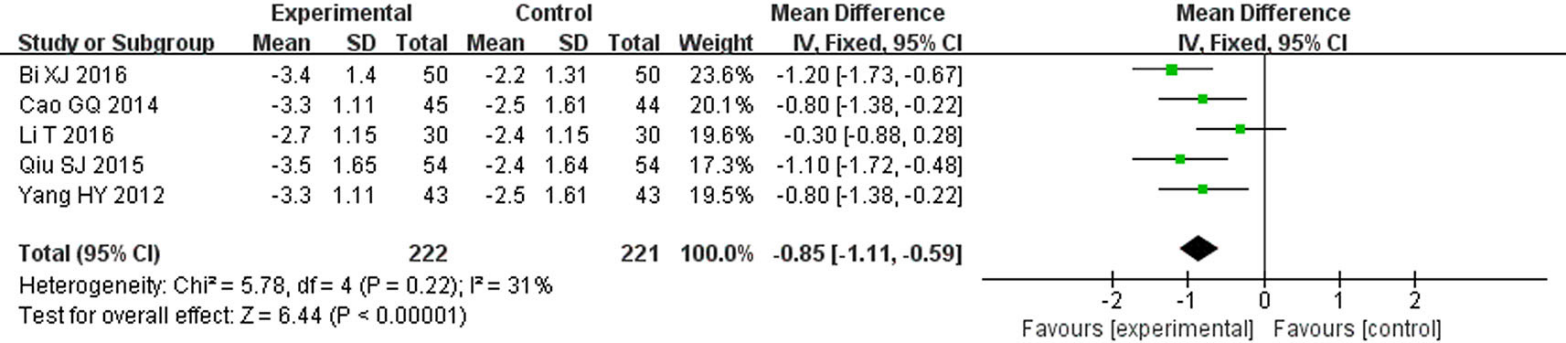
Meta-analysis for comparison of urinary immunoglobulin G (IgG) levels between the experimental and control groups.

Five clinical trials reported urinary IgG levels (Yang et al., 2012; Cao et al., 2014; Qiu, 2015; Bi et al., 2016; Li, 2016). Following the test of heterogeneity (*P* = 0.22, *I^2^* = 31%), a fixed effect model was used to estimate pooled effect size. The results suggested that the level of urinary IgG in STS combined with ARBs was lower than that in the control group. The difference between two groups was statistically significant (MD = −0.85, 95% CI: −1.11 to −0.59; *P* < 0.00001, **Figure 8**).

### Urinary Transferrin (mg/dl)

**Figure 9:**
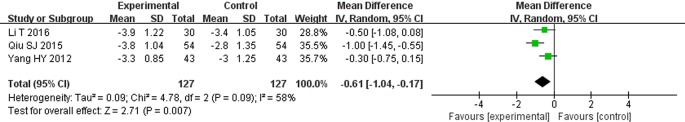
Meta-analysis for comparison of urinary transferrin levels between the experimental and control groups.

Only three clinical trials included urinary transferrin data (Yang et al., 2012; Qiu, 2015; Li, 2016). Following the test of heterogeneity (*P* = 0.09, *I^2^* = 58%), a random effect model was used to estimate pooled effect size. The results suggested that compared with control groups, patients treated with STS combined with ARBs had a significantly lower level of urinary transferrin (MD = −0.61, 95% CI: −1.04 to −0.17; *P* = 0.007, **Figure 9**).

### Blood Pressure Reductions (mmHg)

**Figure 10:**
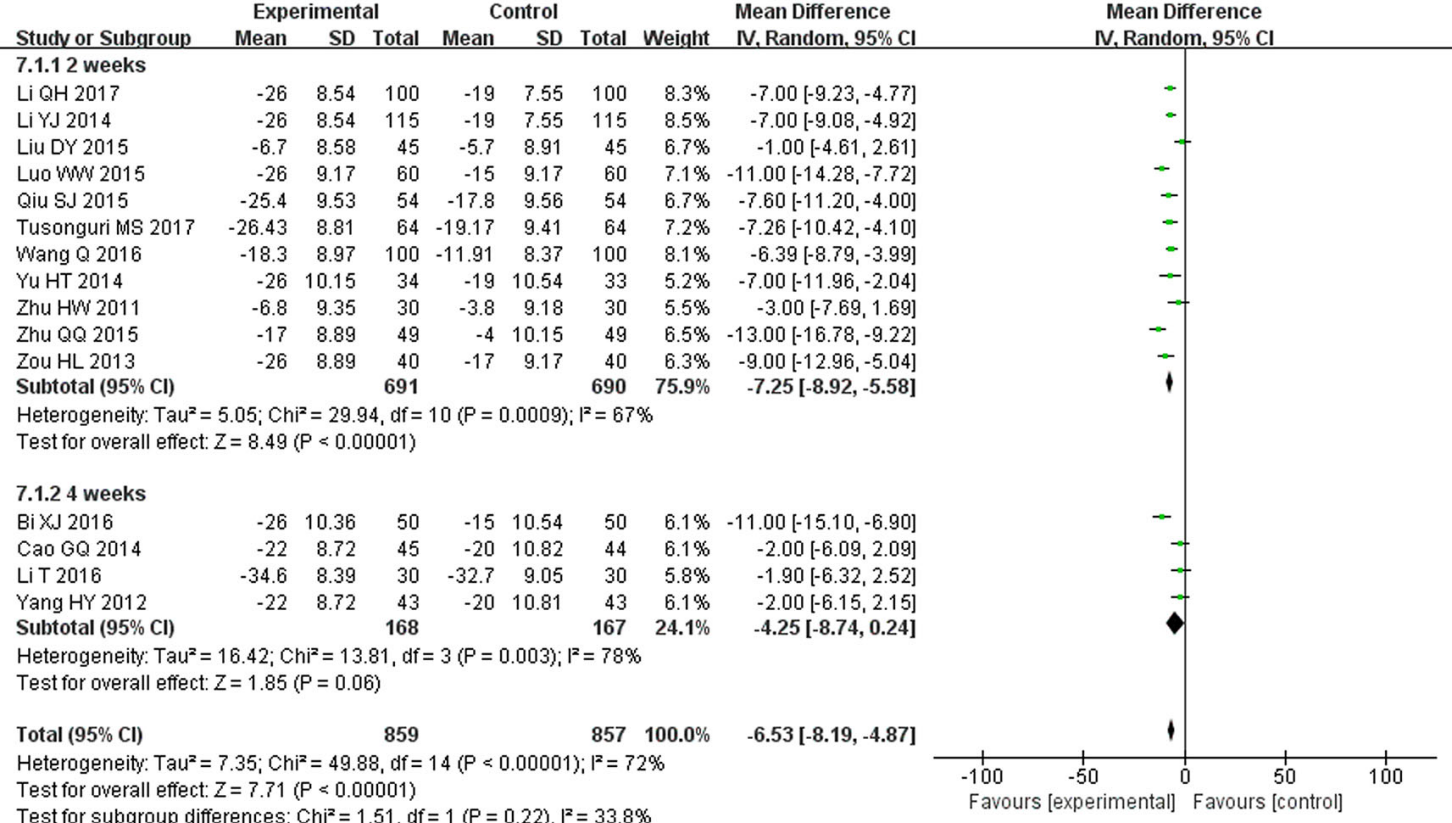
Meta-analysis for comparison of systolic blood pressure (SBP) between the experimental and control groups.

**Figure 11:**
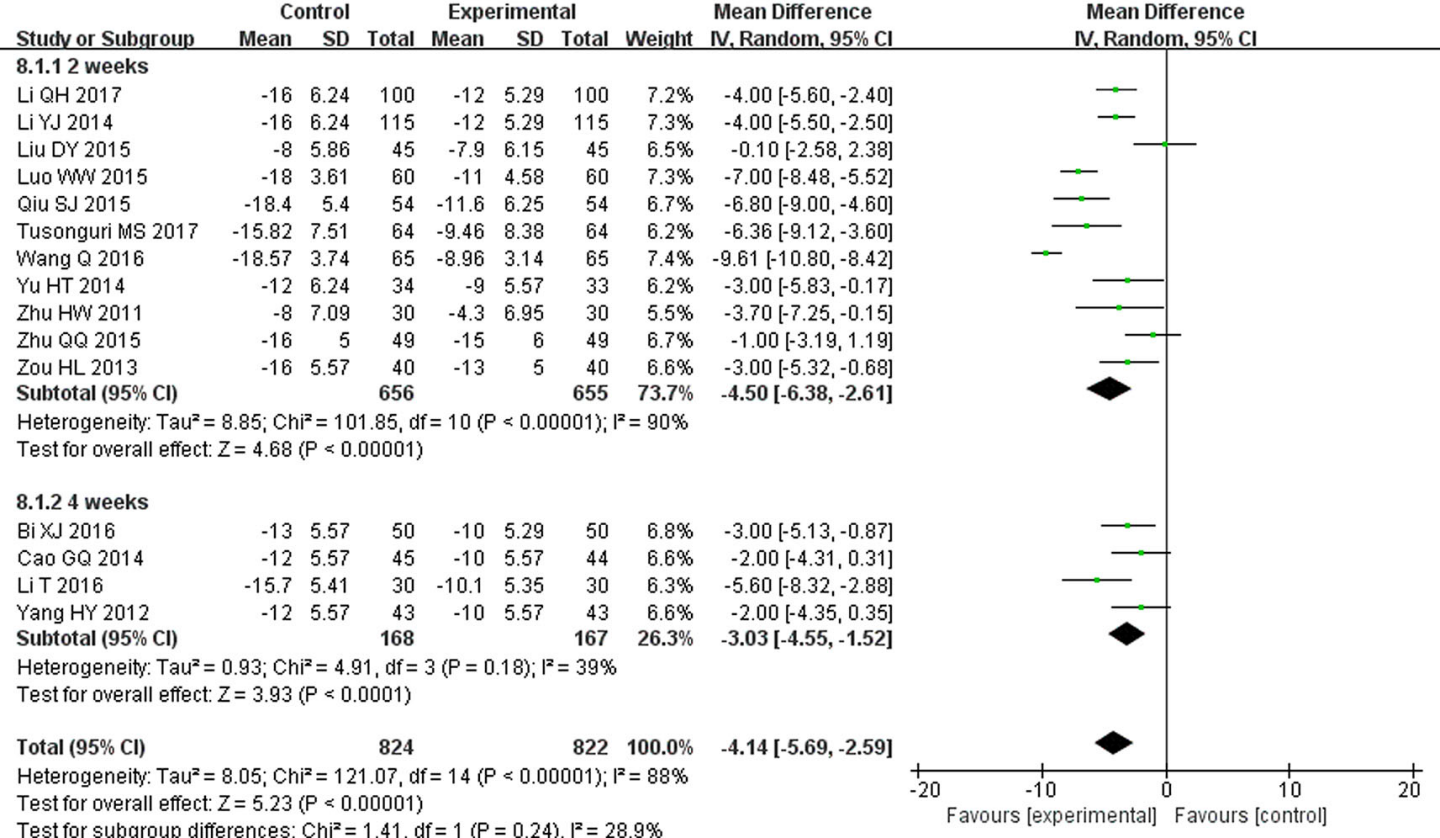
Meta-analysis for comparison of diastolic blood pressure (DBP) between the experimental and control groups.

There were 15 studies that reported SBP and DBP reductions (Zhu et al., 2011; Yang et al., 2012; Zou et al., 2013; Cao et al., 2014; Li, 2014; Yu, 2014; Liu, 2015; Luo, 2015; Qiu, 2015; Zhu, 2015; Bi et al., 2016; Li, 2016; Wang et al., 2016; Li and Guo, 2017; Tusonguri, 2017). Following the test of heterogeneity (*P* < 0.00001 for both SBP and DBP, *I^2^* = 72% and 88% for SBP and DBP, respectively), a random effect model was used to estimate pooled effect size. The results revealed that compared with ARB monotherapy, the combination treatment of STS and ARBs resulted in a significantly greater reduction in SBP (MD = −6.53, 95% CI: −8.19 to −4.87; *P* < 0.00001, **Figure 10**) and DBP (MD = −4.14, 95% CI: −5.69 to −2.59; *P* < 0.00001, **Figure 11**). In subgroup analysis, the hypotensive effect of combined STS and ARBs was better than ARB monotherapy in SBP (MD = −7.25, 95% CI: −8.92 to −5.58; *P* < 0.00001, **Figure 10**) and DBP (MD = −4.50, 95% CI: −6.38 to −2.61; *P* < 0.00001, **Figure 11**) at the 2-week follow-up time point. In addition, the combination treatment also had a significantly greater reduction in DBP (MD = −3.03, 95% CI: −4.55 to −1.52; *P* < 0.0001, **Figure 11**) at the 4-week follow-up time point. However, there were no differences in SBP reduction (MD = −4.25, 95% CI: −8.74 to 0.24; *P* = 0.06, **Figure 10**) at the 4-week follow-up time point.

### Safety

Out of all included studies, only three trials examined adverse events (Zhu et al., 2011; Luo, 2015; Qiu, 2015). These studies all clearly reported that there were no adverse events associated with the STS/ARB combination therapy. However, because the remaining trials did not provide any details regarding adverse effects, it is difficult to draw a conclusion on the safety of STS.

### Subgroup Analysis

To explore the sources of heterogeneity and improve the persuasiveness of the evidence, we conducted a subgroup analysis based on the study characteristics for 24 h urinary protein and SCr, as both of these two biomarkers are crucial indicators that reflect the extent of the renal damage and predict the progression of diseases (Hillege et al., 2002; Lisowska-Myjak et al., 2011). The effect of the combination treatment with STS and ARBs was consistent with the results described above for every subgroup regardless of the duration of treatment, the category of ARBs, the dose of STS, or the number of patients. However, the reduction in 24 h urinary protein did not show significant difference in irbesartan studies (MD = −0.10, 95% CI: −0.25 to −0.04; *P* = 0.17, **Table 2**). Meanwhile, the heterogeneity of each subgroup on 24 h urinary protein declined in various degrees, which indicates that these four factors may be the sources of heterogeneity, especially the intervention course and sample sizes. For SCr, unfortunately, we were not able to remove the heterogeneity from subgroup analysis. However, only Ting Li's study used 4 weeks as the cutoff for treatment duration, which might bias the heterogeneity (Li, 2016). Furthermore, removal of this study from SCr analysis resulted in a considerable reduced *I^2^* (*I^2^* = 54%).

**Table 2:** Subgroup analyses on 24 h urinary protein and SCr.

Factor	24 h urinary protein				SCr			
	N	MD (95% CI)	*P*-value	*I^2^*	N	MD (95% CI)	*P*-value	*I^2^*
Duration								
2 weeks	12	−0.28 (−0.33, −0.23)	<0.00001	14%	10	−23.16 (−24.83, −21.50)	<0.00001	53%
4 weeks	4	−0.14 (−0.20, −0.07)	<0.0001	18%	1	−12 (−16.17, −7.83)	<0.00001	–
Category of ARBs								
Valsartan	11	−0.28 (−0.34, −0.23)	<0.00001	9%	8	−22.66 (−25.25, −20.06)	<0.00001	82%
Irbesartan	2	−0.10 (−0.25, 0.04)	0.17	67%	0	–	–	–
Losartan	3	−0.20 (−0.28, −0.12)	<0.00001	39%	3	−18.15 (−21.54, −14.77)	<0.00001	0%
Dose of STS								
40 mg q.d.	2	−0.15 (−0.25, −0.05)	0.004	0%	2	−18.17 (−22.04, −14.30)	<0.00001	0%
50 mg q.d.	3	−0.21 (−0.38, −0.03)	0.02	0%	2	−22.75 (−24.31, −21.19)	<0.00001	45%
60 mg q.d.	11	−0.25 (−0.30, −0.20)	<0.00001	53%	7	−22.51 (−25.92, −19.09)	<0.00001	84%
Number of patients								
≤100	10	−0.17 (−0.22, −0.12)	<0.00001	11%	6	−19.02 (−23.29, −14.75)	<0.00001	78%
>100	6	−0.33 (−0.39, −0.26)	<0.00001	0%	5	−24.24 (−25.32, −23.17)	<0.00001	22%

SCr, serum creatinine; MD, mean difference; CI, confidence interval; ARB, angiotensin receptor blocker; STS, sodium tanshinone IIA sulfonate; q.d., once daily.

### Sensitivity Analysis

To further confirm the stability of the results of the 24 h urinary protein and SCr, we respectively replaced fixed effect model with random effect model and excluded the most and least weighted trials. The results were not significantly different from those described above, suggesting our meta-analysis results are robust and reliable.

### Publication Bias

**Figure 12:**
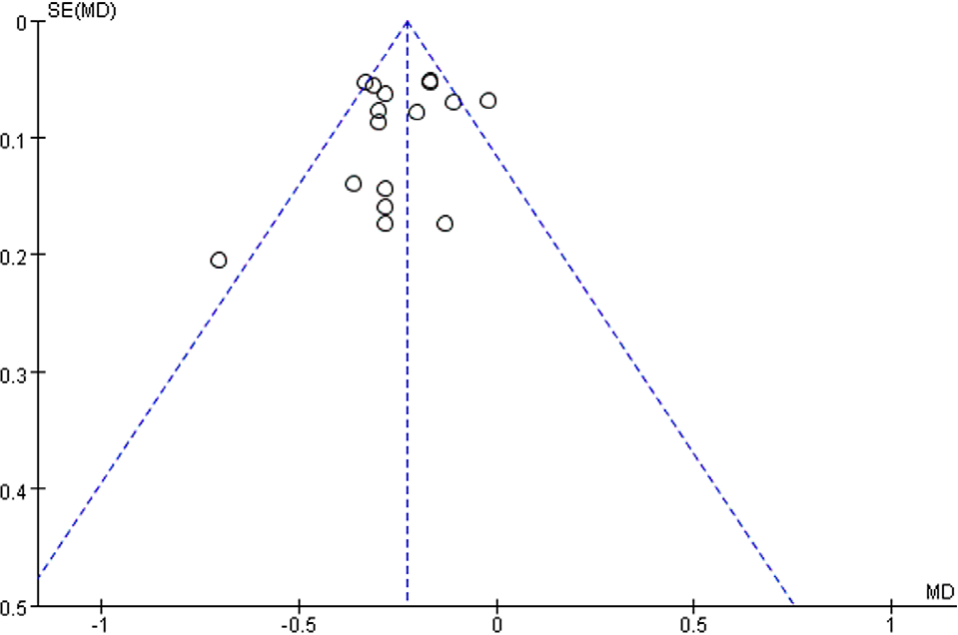
Funnel plot of total 24 h urinary protein.

We assessed the publication bias of 24 h urinary protein with the funnel plot. As shown in **Figure 12**, there was no obvious publication bias in our analysis. However, all the included studies were published in mainland China with positive results, potential publication bias still likely existed.

## Discussion

Although substantial efforts have been made in the prevention and combating of hypertension, it remains one of the most common chronic diseases, and its prevalence is increasing worldwide (Suneel et al., 2011). Hypertension is not only the direct cause of renal damage but can also contribute to the progression of kidney disease (Suneel et al., 2011). The rates of hypertension-associated CKD and ESRD continue to rise, which have a substantial negative influence on public health and health-care financing. BP-lowering therapy, especially the use of the RAAS blockers, has become the main strategy for nephroprotection for patients with renal damage. However, renoprotective effects cannot be achieved to a satisfying extent when these drugs are used alone at the dosages recommended for BP control (Ruggenenti et al., 2008). Thus, other treatments that synergize with RAAS inhibitors to further interfere with events leading to interstitial inflammation and structural damage are desirable (Remuzzi and Bertani, 1998).

Danshen, a popular Chinese herb from dried roots of *S. miltiorrhiza Bunge*, has been used for over 2,000 years for the treatment of cardiovascular diseases without obvious side effects (Yan et al., 2018). Tanshinone IIA, one of the major constituents extracted from Danshen, is officially regarded as a quality control marker as per Chinese Pharmacopoeia (Yan et al., 2018). Sodium tanshinone IIA sulfonate injection is a water-soluble derivative of tanshinone IIA and has become commercially available in China. Previous pharmacological studies have revealed that STS has various biological activities such as anti-atherosclerosis, anti-arrhythmia, improving myocardial blood supply, and cardiac remodeling (Gao et al., 2012). Therefore, STS has been widely used for the management of cardiovascular diseases including angina pectoris and myocardial infarction. Furthermore, intensive research has discovered the renoprotective effect of STS, which has drawn much significant attention and is becoming a research hot spot. Recent publications have demonstrated that tanshinone IIA can exert its renoprotective effects by fighting oxidative stress, attenuating renal fibrosis, regulating inflammation, counteracting ischemia-reperfusion injury, protecting podocytes and endothelial cells, and ameliorating microcirculatory disturbance (Han et al., 2008; Wang et al., 2015; Xu et al., 2016; Cao et al., 2017; Zhu et al., 2017). Increasing evidence indicates that STS combined with antihypertensive drugs like ARBs can additively alleviate the renal dysfunction induced by hypertension. However, the efficacy of STS injection as adjunctive therapy for ARBs on hypertensive nephropathy has not been systematically reviewed and analyzed.

To the best of our knowledge, this is the first systematic review and meta-analysis that assessed renoprotective effects of STS combined with ARBs. A total of 16 trials involving 1,696 patients were identified for this review. According to our analysis of currently available data, we concluded that STS combined with ARBs is more effective than ARB monotherapy in renoprotection, as our findings showed improved eGFR and reduced levels of 24 h urinary protein, SCr, Cys-C, urinary IgG, and urinary transferrin in patients receiving both STS and ARBs compared with those taking only ARBs.

In clinical trials, surrogate end points are often more frequently employed than clinical end points as they are practically measurable. Additionally, use of surrogate end points can reduce the sample size and shorten the duration of studies. Therefore, not surprisingly, we found that surrogate end points were used in all of the analyzed studies, while none of them mentioned primary end points (such as time to doubling of SCr, onset of ESRD, and death). There are a number of reasons for the universality of using surrogate end points in hypertensive nephropathy studies. Firstly, the course of hypertensive nephropathy is long. Clinical outcomes may take years to draw the scientific conclusion. Secondly, as the disease progresses, ARBs may not be suitable for further treatment (such as high creatinine levels and hyperkalemia). Lastly, the use of surrogate end points could aid our understanding of disease processes and mechanisms of action of therapies, directing our decision-making regarding the treatment (Lonn, 2001).

Among multiple surrogate biomarkers that reflect renal function, urinary protein, SCr, and eGFR are the most widely used indicators for the assessment of renoprotective effects of antihypertensive agents in clinical trials. After the pioneering Ramipril Efficacy In Nephropathy (REIN) trials found that short-term reduction in proteinuria slows the progression to ESRD in the long term, the reduction of proteinuria has been considered as a novel target of renoprotective therapy (The GISEN Group, 1997). Subsequent studies confirmed that proteinuria is a potent biomarker of renal dysfunction, and the slowest progression was observed in patients with the lowest residual proteinuria (Ruggenenti et al., 2003). Therefore, the 7th Report of the Joint National Committee on Prevention, Detection, Evaluation, and Treatment of High Blood Pressure (JNC 7) and the National Kidney Foundation (NKF) guidelines recommend that reduction of albuminuria and BP should be included as the cornerstone in a treatment strategy for hypertensive nephropathy (Ruggenenti et al., 2008; Hart and Bakris, 2010). Furthermore, albuminuria has also been identified as a risk factor for cardiovascular events. Consequently, special attention should be paid to the degree of proteinuria when evaluating the improvement of renal function. In our study, we chose the 24 h urinary protein as one of the major outcomes. Based on 16 trials, the combination treatment of STS and ARBs had better efficacy in terms of preventing the progression of proteinuria. However, subgroup analysis covering three different categories of ARBs found no difference in the irbesartan group. This might be attributable to the small number of patients in the irbesartan group. Thus, further trials in a large series are required to determine the efficacy of STS plus irbesartan. SCr, an accurate index for renal function, also reliably predicts the risk for renal injury (Spanaus et al., 2010; Yan et al., 2014). The results of our analysis indicated that STS combined with ARBs can significantly decrease serum levels of creatinine. However, it should be acknowledged that there was significant heterogeneity despite subgroup analysis. The fact that creatinine is influenced by various factors such as age, gender, and diet that may bias the result should also not be ignored. Thus, in recent clinical guidelines, eGFR, calculated based on the SCr level and other parameters, is recommended for the estimation of renal function because of its sensitivity and high specificity for one-time measures of renal damage or dysfunction (Qaseem et al., 2013; Stevens and Levin, 2013). Previous studies have demonstrated that angiotensin converting enzyme inhibitors (ACEIs) and ARBs could maintain eGFR during the progress of hypertension (Cheng et al., 2016). Similar to other outcomes, we found that STS and ARB combination is more effective than ARB monotherapy in improving eGFR. However, there is yet no consensus among different guidelines on which formula to use for calculating eGFR, possibly leading to enormous calculation difference due to the use of different formulas. Although our results showed no heterogeneity, none of the included studies in this review reported the equation used for eGFR calculation, which may increase the risk of bias.

Cys-C is another biomarker that detects renal damage, especially in the early stage of nephropathy. Unlike SCr, urinary IgG, or transferrin, Cys-C is not influenced by gender, race, and muscle mass, and thus has recently drawn considerable attention (Peralta et al., 2011). Cys-C also exhibits predictive value for the prognosis of renal and cardiovascular diseases (Peralta et al., 2011; Ozkok et al., 2014; Barr et al., 2017; Zhang et al., 2017). In our study, we found that STS plus ARBs therapy was better than ARBs alone in improving urinary protein, SCr, and eGFR, which also suggested that STS could be beneficial as an “add-on” medication for hypertensive patients.

In addition, systolic and diastolic BP reductions were evaluated to detect the effects of STS in terms of BP control. The results revealed that STS/ARB combination decreased the average SBP and DBP by 6.53 and 4.14 mmHg, respectively, compared with ARB monotherapy. STS provided auxiliary hypotensive effects, thereby enabling hypertensive patients to better achieve their target BP goals. In the subgroup analysis according to the intervention course, SBP reductions were statistically significant when 2 weeks was used as the cutoff for intervention course. However, there were no statistical differences when 4 weeks was used in the analysis. Notably, the majority of patients enrolled in this systematic review had reached the target SBP and DBP by the end of 2 weeks. Therefore, according to above results, we speculate that STS as an adjuvant treatment may not have further effects on SBP when SBP is already under control. However, there were only four trials where combined therapy lasted for 4 weeks (Yang et al., 2012; Cao et al., 2014; Bi et al., 2016; Li, 2016). Although the results did not show significant differences, SBP had a tendency to be lower in the STS/ARB combination treatment group at 4 weeks. Conducting more trials may alter the statistical results. Meanwhile, we recognized that there was considerable heterogeneity. We attempted to address this heterogeneity by carefully checking all of the included studies and performing sensitivity analysis and several subgroup analyses. But, unfortunately, we were not able to figure out the origin of the heterogeneity. We believe it may be caused by several reasons, e.g., different timing of medication and BP measurement, different types of ARBs and dosages of STS used, etc., which might bias the results. Accordingly, these results should be interpreted cautiously.

## Limitations

This study has several limitations. Firstly, all included trials were published in Chinese, and only three trials provided specific information of random sequence generation (Li, 2014; Qiu, 2015; Li and Guo, 2017). We tried to contact the authors by telephone, fax, or e-mail to confirm, but received no replies. Additionally, all included trials didn't provide details of allocation concealment, blinding of participants and personnel, and blinding of outcome assessment, which, as shown in **Figure 3**, can lead to high risk of selection, performance, and detection bias respectively. The absence of allocation concealment and blinding may subjectively influence participants and researchers as well, although objective outcomes are enrolled in all the included studies. In view of these methodological problems, in order to achieve more conclusive results, we suggest that future clinical trials in large series should adopt such methods as proper randomization, allocation concealment, participant and personnel blinding, and assessment blinding. Secondly, the treatment course in the 16 trials was short: either 2 or 4 weeks. Therefore, we were unable to assess the long-term efficacy of STS. Thirdly, the actual clinical evidence such as doubling of SCr, progression to dialysis, and death are lacking. Large-scale long-term studies should be conducted to evaluate renoprotective effects of STS. Lastly, special attention should be paid to adverse drug events or reactions. Safety is a fundamental principle in the provision of herbal products for health care. However, there is insufficient evidence to draw a conclusion on the issue of safety in this review. Because 13 trials did not report about adverse drug events or reactions. Current studies regarding the factors of adverse effect caused by STS often focus on allergic reactions. Although STS is extracted by *S. miltiorrhiza Bunge* with high purity, the composition is complex, which may lead to adverse events. Clinicians should dilute the injection according to the package insert strictly and avoid mixed application to prevent adverse events happening.

## Conclusions

In conclusion, this meta-analysis included 16 RCTs in assessing the effect of STS plus ARBs on kidney-related outcomes in patients with hypertensive nephropathy compared to ARB monotherapy. The results illustrated that STS combined with ARBs not only exerts auxiliary antihypertensive effects but also protects renal function. These results, however, should be interpreted with caution due to the limitations described above. Further large-scale, multicenter, long-term, randomized, and double-blind clinical trials are needed. Meanwhile, the safety of STS should also be evaluated.

## Data Availability Statement

All datasets generated for this study are included in the article/**Supplementary Material**.

## Author Contributions

JX and CZ conceived the study, conducted the database search, assessed included studies, extracted the data, and wrote the paper. XS checked the data with JX and CZ. JL, ML, and WJ conducted the data analysis. ML, WJ, and ZF revised the final manuscript.

## Funding

This work was supported by the National Natural Science Foundation of China (grant 81573909), the Youth Natural Science Foundation of Jiangsu Province (grant BK20181095), and the Social Development Key Programs of Science and Technology Commission Foundation of Jiangsu Province (grant BE2015730).

## Conflict of Interest

The authors declare that the research was conducted in the absence of any commercial or financial relationships that could be construed as a potential conflict of interest.
