# Supplementary material for: Efficacy and Safety of Sodium Tanshinone IIA Sulfonate Injection on Hypertensive Nephropathy: A Systematic Review and Meta-Analysis
Source: Front Pharmacol. 2019 Dec 24;10:1542. doi: 10.3389/fphar.2019.01542 (PMC6937217; doi:10.3389/fphar.2019.01542)
Supplement: Supplementary file 1 [file DataSheet_1.docx]

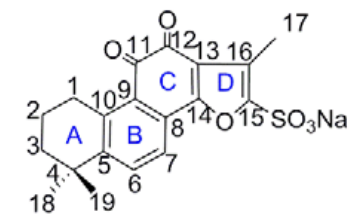


**FIGURE 1|** The structure of sodium tanshinone ⅡA sulfonate.


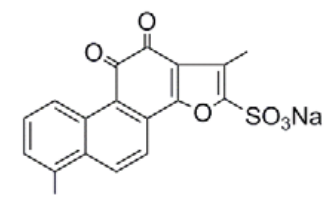


**FIGURE 2|** The structure of sodium tanshinone Ⅰ sulfonate.


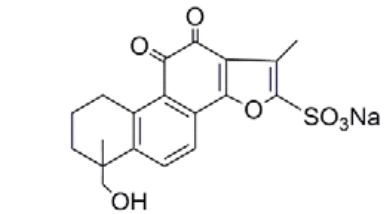


**FIGURE 3|** The structure of sodium tanshinone ⅡB sulfonate.


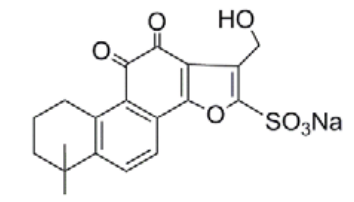


**FIGURE 4|** The structure of sodium przewaquinone A sulfonate.
