# Supplementary material for: Efficacy and Safety of Sodium Tanshinone IIA Sulfonate Injection on Hypertensive Nephropathy: A Systematic Review and Meta-Analysis
Source: Front Pharmacol. 2019 Dec 24;10:1542. doi: 10.3389/fphar.2019.01542 (PMC6937217; doi:10.3389/fphar.2019.01542)
Supplement: Supplementary file 2 [file Table_1.docx]

| Study | Source | Species, concentration | Quality control reported? (Y/N) | Chemical analysis reported? (Y/N) |
| --- | --- | --- | --- | --- |
| Tusonguri, 2017 | Unclear | *Salvia miltiorrhiza Bge* (Sodium Tanshinone ⅡA Sulfonate, 5mg/ml) | Y - Prepared according to Chinese pharmacopeia | Y – HPLC^*^ |
| Li and Guo, 2017 | Shanghai No.1 Biochemical & Pharmaceutical Co., Ltd. | *Salvia miltiorrhiza Bge* (Sodium Tanshinone ⅡA Sulfonate, 5mg/ml) | Y - Prepared according to Chinese pharmacopeia (H31022558) | Y – HPLC^*^ |
| Wang et al., 2016 | Shanghai No.1 Biochemical & Pharmaceutical Co., Ltd. | *Salvia miltiorrhiza Bge* (Sodium Tanshinone ⅡA Sulfonate, 5mg/ml) | Y - Prepared according to Chinese pharmacopeia (H31022558) | Y – HPLC^*^ |
| Li, 2016 | Shanghai No.1 Biochemical & Pharmaceutical Co., Ltd. | *Salvia miltiorrhiza Bge* (Sodium Tanshinone ⅡA Sulfonate, 5mg/ml) | Y - Prepared according to Chinese pharmacopeia (H31022558) | Y – HPLC^*^ |
| Bi et al., 2016 | Unclear | *Salvia miltiorrhiza Bge* (Sodium Tanshinone ⅡA Sulfonate, 5mg/ml) | Y - Prepared according to Chinese pharmacopeia | Y – HPLC^*^ |
| Zhu, 2015 | Unclear | *Salvia miltiorrhiza Bge* (Sodium Tanshinone ⅡA Sulfonate, 5mg/ml) | Y - Prepared according to Chinese pharmacopeia | Y – HPLC^*^ |
| Qiu, 2015 | Shanghai No.1 Biochemical & Pharmaceutical Co., Ltd. | *Salvia miltiorrhiza Bge* (Sodium Tanshinone ⅡA Sulfonate, 5mg/ml) | Y - Prepared according to Chinese pharmacopeia (H31022558) | Y – HPLC^*^ |
| Luo, 2015 | Shanghai No.1 Biochemical & Pharmaceutical Co., Ltd. | *Salvia miltiorrhiza Bge* (Sodium Tanshinone ⅡA Sulfonate, 5mg/ml) | Y - Prepared according to Chinese pharmacopeia (H31022558) | Y – HPLC^*^ |
| Liu, 2015 | Shanghai No.1 Biochemical & Pharmaceutical Co., Ltd. | *Salvia miltiorrhiza Bge* (Sodium Tanshinone ⅡA Sulfonate, 5mg/ml) | Y - Prepared according to Chinese pharmacopeia (H31022558) | Y – HPLC^*^ |
| Yu, 2014 | Unclear | *Salvia miltiorrhiza Bge* (Sodium Tanshinone ⅡA Sulfonate, 5mg/ml) | Y - Prepared according to Chinese pharmacopeia | Y – HPLC^*^ |
| Li, 2014 | Unclear | *Salvia miltiorrhiza Bge* (Sodium Tanshinone ⅡA Sulfonate, 5mg/ml) | Y - Prepared according to Chinese pharmacopeia | Y – HPLC^*^ |
| Cao et al., 2014 | Unclear | *Salvia miltiorrhiza Bge* (Sodium Tanshinone ⅡA Sulfonate, 5mg/ml) | Y - Prepared according to Chinese pharmacopeia | Y – HPLC^*^ |
| Zou et al., 2013 | Shanghai No.1 Biochemical & Pharmaceutical Co., Ltd. | *Salvia miltiorrhiza Bge* (Sodium Tanshinone ⅡA Sulfonate, 5mg/ml) | Y - Prepared according to Chinese pharmacopeia (H31022558) | Y – HPLC^*^ |
| Wu, 2013 | Shanghai No.1 Biochemical & Pharmaceutical Co., Ltd. | *Salvia miltiorrhiza Bge* (Sodium Tanshinone ⅡA Sulfonate, 5mg/ml) | Y - Prepared according to Chinese pharmacopeia (H31022558) | Y – HPLC^*^ |
| Yang et al., 2012 | Unclear | *Salvia miltiorrhiza Bge* (Sodium Tanshinone ⅡA Sulfonate, 5mg/ml) | Y - Prepared according to Chinese pharmacopeia | Y – HPLC^*^ |
| Zhu et al., 2011 | Shanghai No.1 Biochemical & Pharmaceutical Co., Ltd. | *Salvia miltiorrhiza Bge* (Sodium Tanshinone ⅡA Sulfonate, 5mg/ml) | Y - Prepared according to Chinese pharmacopeia (H31022558) | Y – HPLC^*^ |

^*^Sodium Tanshinone ⅡA Sulfonate > 95%, other ingredients (Sodium Tanshinone ⅡB Sulfonate, Sodium Tanshinone Ⅰ Sulfonate, Sodium Przewaquinone A Sulfonate) < 5%
